# Implementing daily chlorhexidine gluconate treatment for the prevention of healthcare-associated infections in non-intensive care settings: A multiple case analysis

**DOI:** 10.1371/journal.pone.0232062

**Published:** 2020-04-24

**Authors:** Jackson S. Musuuza, Tonya J. Roberts, Ann Schoofs Hundt, Pascale Carayon, Michele L. Zimbric, Valeri Schuetz, Mel Reppen, Windy Smith, Kirsten Koffarnus, Roger L. Brown, Jason Bowling, Kat Jalali, Nasia Safdar

**Affiliations:** 1 Department of Medicine, University of Wisconsin School of Medicine and Public Health, Madison, WI, United States of America; 2 William S. Middleton Memorial Veterans Hospital, Madison, WI, United States of America; 3 School of Nursing, University of Wisconsin-Madison, Madison, WI, United States of America; 4 Center for Quality and Productivity Improvement, Wisconsin Institute for Healthcare Systems Engineering, University of Wisconsin-Madison, Madison, WI, United States of America; 5 Department of Industrial and Systems Engineering, University of Wisconsin-Madison, Madison, WI, United States of America; 6 SSM Health, St. Mary’s Hospital, Madison, WI, United States of America; 7 American Family Children's Hospital, Madison, WI, United States of America; 8 University of Texas Health Science Center, San Antonio, TX, United States of America; University of Minnesota, UNITED STATES

## Abstract

**Introduction:**

Daily bathing with chlorhexidine gluconate (CHG) in hospitalized patients reduces healthcare-associated bloodstream infections and colonization by multidrug-resistant organisms. Achieving compliance with bathing protocols is challenging. This non-intensive care unit multicenter project evaluated the impact of organizational context on implementation of CHG and assessed compliance with and healthcare workers’ perceptions of the intervention.

**Materials and methods:**

This was a multiple case study based on the SEIPS (Systems Engineering Initiative for Patient Safety) model of work system and patient safety. The four sites included an adult cardiovascular unit in a community hospital, a medical-surgical unit in an academic teaching pediatric hospital, an adult medical-surgical acute care unit and an adult neuroscience acute care unit in another academic teaching hospital. Complementary data collection methods included focus groups and interviews with healthcare workers (HCWs) and leaders, and direct observations of the CHG treatment process and skin swabs.

**Results:**

We collected 389 bathing observations and 110 skin swabs, conducted four focus groups with frontline workers and interviewed leaders. We found variation across cases in CHG compliance, skin swab data and implementation practices. Mean compliance with the bathing process ranged from 64% to 83%. Low detectable CHG on the skin was related to immediate rinsing of CHG from the skin. Variation in the implementation of CHG treatments was related to differences in organizational education and training practices, feedback and monitoring practices, patient education or information about CHG treatments, patient preferences and general unit patient population differences.

**Conclusion:**

Organizations planning to implement CHG treatments in non-ICU settings should ensure organizational readiness and buy-in and consider delivering systematic and ongoing training. Clear and systematic implementation policies across patients and units may help reduce potential confusion about treatment practices and variation across HCWs. Patient populations and unit factors need to be carefully considered and procedures developed to manage unique challenges.

## Introduction

Healthcare-associated infections (HAIs) are the most frequent adverse events in healthcare worldwide, causing significant mortality, morbidity and financial burden [1–4]. Daily bathing or treatment of hospitalized patients with the broad-spectrum antiseptic chlorhexidine gluconate (CHG) has been shown to reduce healthcare-associated bloodstream infections [5–7] and colonization by multidrug resistant organisms, particularly in the intensive care unit (ICU) [5]. Extending this treatment outside the ICU may have benefit as well, particularly for patients with medical devices, such as central lines, midline catheters and lumbar drains [8]. However, there is considerable variation in the implementation of CHG treatment when deployed outside the ICU. In order for CHG treatment to have a beneficial effect, high-fidelity implementation of CHG treatment is essential [9]; yet there is a paucity of data on CHG implementation in diverse care settings, particularly the impact of contextual factors such as those related to organizational factors (e.g., nurse staffing levels and CHG supply) [10, 11].

Using a multiple case study design [12], we undertook a multisite study using mixed methods to examine the impact of organizational context on CHG treatment implementation and use in multiple non-ICU settings and populations. We assessed compliance with daily CHG treatment, evaluated healthcare workers’ (HCWs) perceptions of the intervention and identified barriers and facilitators to implementation of CHG treatment in a variety of organizational contexts.

## Materials and methods

### Study design

We employed a multiple case study design using mixed methods for data collection and analysis [13]. We define a “case” as a hospital unit in which CHG treatment was implemented. We chose the case study approach because it is particularly useful to gain an in-depth appreciation of an issue, event or phenomenon of interest in its naturalistic context (in our case, the implementation of CHG treatment) [14, 15]. The case study approach lends itself well to capturing information on 'how', 'what' and 'why' questions, such as 'How is the intervention implemented and perceived by healthcare workers?' [16]

In order to get an in-depth understanding of contextual factors, this project was guided by the Systems Engineering Initiative for Patient Safety (SEIPS) [17] model, which describes context as various aspects of systems that interact to influence patient safety [17–22]. The SEIPS model focuses on five elements of the work system—person, tasks, tools and technologies, physical environment and organizational conditions. These interact to affect care processes (e.g., patient CHG treatment), which then affect patient and organizational outcomes, such as HCW, patient and caregiver engagement, safety, compliance, workload and efficiency.

We used the following multiple data collection methods: focus group discussions and interviews of HCWs, direct observations of CHG treatment process and microbiologic assessment of CHG concentration on the skin.

### Setting and samples

Four non-ICUs (i.e., four cases) from three study sites (i.e., hospitals) participated in our project. This included: an adult cardiovascular unit of a community hospital (case A), a medical/surgical unit in an academic teaching pediatric hospital (case B), an adult medical/surgical acute care unit of an academic teaching hospital (case C) and an adult neuroscience acute care unit of the same academic teaching hospital as case C (case D). Units C and D used CHG in an indications-based way (e.g., patients with lines and those going for surgery), while Units A and B had a blanket policy where CHG treatment was provided to all patients on the unit. The CHG bathing protocols used at different facilities in this study were more elaborate than other more targeted uses of CHG (e.g., cleaning around lines and devices). Specifically, the protocols involved the following steps: gathering the necessary supplies, performing hand hygiene, donning gloves, applying at least two pumps of CHG soap to each washcloth, using one CHG washcloth per body part, rinsing each CHG-bathed area with a new washcloth and applying a CHG-compatible lotion. All sites used washcloths with CHG soap (Hibiclens® 4%), and none used the no-rinse pre-packaged 2% CHG wipes. Details of the CHG treatment protocol can be found in a CHG treatment toolkit published by our group at https://www.hipxchange.org/CHGBathing. Characteristics of the four units and study participants are provided in Table 1. The terms “case” and “unit” are used interchangeably throughout the paper.

**Table 1:** Characteristics of participating units and participants.

	Adult Medical/Surgical Cardiovascular Care Unit (Case A)	Pediatric Medical/Surgical Unit (Case B)	Adult Medical/ Surgical Unit (Case C)	Adult Neuroscience Unit (Case D)
**Total number of beds at hospital**	418	89	644	
**Number of beds on unit**	30	24	45	39
**Average occupancy on unit (%)**	83%	85%	74%	95%
**HCWs performing CHG-treatment**	4 CNAs during day shift	Varies between 3–4 RNs & 1–2 NAs per shift	5–6 health techs per shift	4–5 health techs per shift
**Description of CHG treatment training**	Research team provided on-site introduction to unit leadership council; training then provided to HCWs; unit nurse manager followed up with those unable to attend; minutes of training posted on unit.	Training about CHG was provided during weekly staff meetings a month prior to implementing CHG treatment. The hospital’s infection preventionist conducted the training. Staff were educated about the steps involved in the CHG treatment protocol and were also given written material covering the research behind CHG treatment and frequently asked questions.	Research team shared training materials; unit nurse educator instructed HCWs on procedure in morning huddle and email; those unable to participate in huddle communicated with one-on-one during “rounding in service.”	Research team shared training materials; unit nurse educator instructed HCWs on procedure through training sessions and email.
**Focus group:**				
• # of participants (gender)	• 5 (all female)	• 6 (all female)	• 5 (4 female; 1 male)	• 4 (2 female; 2 male)
• length	• 70 minutes	• 65 minutes	• 53 minutes	• 53 minutes
**Interviews:**				
• # conducted (people interviewed)	• 2 (director infection prevention, unit nurse manager; all female)	• 2 (two infection preventionists (paired interview), unit nurse manager; all female)	• 3* (hospital epidemiologist with director of Infection Prevention, director Nurse Center of Excellence, unit nurse educator; 3 female, 1 male)	
• total length	• 75 minutes	• 99 minutes	• 161 minutes	
**Number of observations**	179	110	54	46
**Number of patients swabbed**	39	58	8	5

* Interviews for cases C and D included same hospital epidemiologist with director of Infection Prevention (paired interview) and director of Nurse Center of Excellence; interviews addressed both cases.

### Direct observations

The data collection process for the CHG treatment observations has been described in detail elsewhere [23]. Briefly, trained observers conducted direct observations of HCWs giving CHG treatments to determine the level of compliance with the steps in the CHG process. Observation data were collected using a checklist similar to our previous study [24] and directly entered into REDCap 8.1.1^®^, a real-time online data collection platform [25]. We analyzed direct observation data by conducting descriptive analyses to assess completion of CHG treatment checklist items and the duration of the CHG treatment.

### Skin swabs

To assess compliance with the CHG treatment process, we measured the concentration of CHG on patients’ skin. We sampled three anatomical sites one hour and 24 hours after the CHG treatment by holding a swab vertical to the skin surface and rubbing the swab across a 25 cm^2^ surface area of intact skin. Anatomic sites sampled included the neck from jawline to clavicle, antecubital fossae and axillary area. We measured CHG concentration using a semi-quantitative colorimetric assay described previously [26, 27]. We conducted descriptive statistics to determine the proportion of patients with any detectable CHG on the three sites at the two time intervals. This analysis was conducted using Stata^®^ version 14 (Stata Corp, College Station, Texas, USA).

### Focus groups and interviews

#### Data collection

HCWs primarily responsible for performing CHG treatments voluntarily participated in focus groups. They were informed about the study during staff meetings and were invited to participate in focus groups as their schedule allowed. We provided a description of the project, an overview of the work system component of the SEIPS model [17, 28], and a review of the standard process for performing CHG treatments (Table 2). Beginning with the first step in the CHG process–gathering supplies–the participants described how they completed each step and identified any system barriers and facilitators they faced in successfully accomplishing the step [29]. The focus group prompts were broadly informed by the SEIPS model to address various aspects of the work system. Six to ten months after CHG treatment was implemented, two researchers conducted interviews with the nurse manager (NM) or nurse educator (NE) for each of the four units, as well as an infection preventionist (IP). A nursing management leader for Units C & D (the same hospital) was also interviewed. These interviews captured organizational practices prior to and after the intervention was implemented. Interview questions were focused on several important aspects of implementing and maintaining the CHG bathing initiative, including prioritizing the initiative, communicating about the initiative to stakeholders, training, monitoring compliance and barriers and facilitators to implementation or maintenance. Interview guides are available as supporting information (S1 and S2 Files). We audio recorded and transcribed all interviews and focus groups.

**Table 2:** Chlorhexidine treatment process compliance for the four cases (study sites).

	Case A	Case B	Case C	Case D
**Number of observations**	179	110	54	46
**Mean duration of bath (minutes)**	12.7 (SD = 0.8)	11.8 (SD = 0.8)	13.4 (SD = 3.5)	16.7 (SD = 2.8)
**A. Gather Supplies (% yes)**				
Basin or Ziploc Bag	95.5%	98.2%	98.1%	82.6%
Washcloths	100%	100%	100%	93.5%
CHG soap	100%	100%	98.1%	95.7%
CHG compatible lotion	91.1%	79.1%	7.4%	6.5%
Patient or family education about CHG	61.5%^a^	55.5%^a^	N/A	N/A
**B. Hand hygiene (% yes)**				
Hand hygiene performed	83.2%	91.8%	83.3%	87.0%
Don clean gloves	92.3%	100%	87.0%	100%
Personal Protective Equipment	66.7%	98.1%	57.8%	12.5%
**C. Perform CHG Treatment (% yes)**				
Wet washcloths	98.3%	100%	100%	95.7%
Wash patient's face with non-CHG soap and water	86.0%	93.0%	81.5%	69.6%
Use 1 washcloth to wash each body part	75.0%	42.7%	81.5%	43.5%
Apply 2 pumps of CHG to each washcloth	98.3%	86.4%	61.1%	54.3%
Use different clean wet washcloth to rinse CHG off body part	52.5%	32.1%	85.2%	50.0%
Use non-CHG soap and water on genital area/perineum	91.6%	85.4%	87.0%	89.1%
Rinse genital area/perineum with clean wet washcloths	57.0%	62.1%	85.2%	56.5%
Avoid CHG soap on drains, lines, and/or dressings	94.4%	97.9%	74.1%	45.7%
Towel dry skin	99.0%	100%	94.4%	91.3%
Apply Medline or Aloe Vesta lotion	75.4%	55.1%	11.1%	4.3%
Complete CHG treatment with no skin below jaw line missed	64.8%	64.0%	59.3%	65.2%

Denominator in calculations excludes cases where the step was not applicable, for example, the denominator for “Avoid CHG soap on drains, lines, and/or dressings” excludes patients who did not have IV lines, drains or dressings.

“a” = Calculated only for baths that were not first baths. N/A = None of the baths were first baths, hence patient or family education about CHG was not observed.

#### Data analysis

Content analysis was used to code the qualitative data. Two researchers coded the data according to a predetermined multidimensional coding scheme based on the SEIPS model. Specifically, the data were coded to describe first how HCW bathing practices corresponded with recommended procedures, then the barriers and facilitators that influenced bathing practices at each step were coded. The two researchers convened and compared coding, discussing discrepancies until they reached agreement.

### Ethics

The research was determined to be exempt by the University of Wisconsin Health Sciences Institutional Review Board because it was a quality improvement project. However, we obtained informed verbal consent from all the participants before any data was collected.

## Results

We performed 389 direct observations of the CHG treatment process (179 on Unit A, 110 on Unit B, 54 on Unit C and 46 on Unit D) and collected skin swabs from 110 patients (Table 1). Unit A had the highest mean overall compliance of 83% (SD = 16%) and the lowest mean duration of CHG treatment of 12.7 (SD = 0.8) minutes. Unit D had the lowest mean overall compliance of 64% (SD = 31%) and the longest mean duration of CHG treatment of 16.7 (SD = 2.8) minutes. For Unit A, using one washcloth for each body part was the most missed checklist item, occurring in 53% of the observed CHG treatments. For Unit D, the CHG bathing processes with the lowest compliance included: 1) applying a moisturizing lotion (4%), and 2) use of PPE when applicable (13%). The mean proportion of patients with detectable CHG 1 hour and 24 hours post CHG treatment for Unit A was 70% (SD = 10%) and 59% (SD = 7%), respectively.

Units C and D had the highest mean proportion of patients with detectable CHG 1 hour and 24 hours post CHG treatment at 80% (SD = 17%) and 84% (SD = 8%), respectively. Detectable CHG was lowest in Unit B (1 hour: 57%, SD = 2% and 24 hours: 22%, SD = 6%). Results for observations and swab data for all the 4 Units are summarized in Tables 2 and 3.

**Table 3:** Proportion of patients with ANY detectable CHG concentration post-CHG treatment.

	Patients with any detectable CHG 1 hour post CHG treatment, n/N* (%)				Patients with any detectable CHG 24 hours post CHG treatment, n/N* (%)			
	Neck area	Antecubital fossa	Axilla	Mean proportion with detectable CHG (SD)	Neck area	Antecubital fossa	Axilla	Mean proportion with detectable CHG (SD)
**Case A**	23/39 (59%)	31/39 (79%)	28/39 (72%)	70% (10%)	21/39 (54%)	26/39 (67%)	22/39 (56%)	59% (7%)
**Case B**	32/58 (55%)	34/58 (58%)	30/58 (58%)	57% (2%)	13/58 (22%)	16/58 (28%)	10/58 (17%)	22% (6%)
**Cases C & D**	10/13 (77%)	12/13 (92%)	11/13 (85%)	84% (8%)	7/10 (70%)	10/10 (100%)	7/10 (70%)	80 (17%)

*n/N = number of patients with any detectable CHG/total number of patients swabbed

To learn about the facilitators and barriers to CHG implementation in the various units, for each case, we conducted 4 focus groups with frontline HCWs who performed the CHG treatments and 8 interviews with representatives from hospital administration, infection prevention and unit leadership. Verbatim illustrative quotations (Q) from the focus groups and interviews are presented in Table 4. Results are described below, organized thematically.

**Table 4:** Representative quotes.

Illustrative quotations (Q)	Theme
**Q1:** *Interviewer*: Are there factors that when you implemented this process that you think made it tough for people to successfully accomplish this, you know, the process as we're suggesting? *Nurse Educator*: I think we have a lot of different education going out at the same time oftentimes. It's not just this one thing. I, I think, we have a new early mobility project that I'm educating on right now. Then we had immunizations, and then we had a new discharge note, and then we had a, let's see, what else? It, it just seems like, and it's just, you know, we are the face of change for univer-, for our area. I mean, other hospitals are going to follow what we do.	Variations in HCW training
**Q2:** Well, I personally don’t educate people on the Hibiclens. I just literally give them a Hibiclens bath, and then unless they really ask, because then I go into the details…	Inconsistent patient and family education
**Q3:** … when it comes to the babies, like the two-month, like the three- to four-months, like I sometimes use one washcloth, but I'll fold it in half, because I think it's a waste. I think I'm wasting washcloths …	Deviations from CHG treatment steps
**Q4:** Because everybody does everything different.	Deviations from CHG treatment steps
**Q5:** … we don’t have that special lotion …to use after. I don’t want people get ideas to put lotion on, on top of CHG because we don’t have that… I tell them not to put anything. Because we have, you know, it’s different, acute unit… You have a lot of people is like, I want my, use my own lotion… And then, that’s where you get …lose the battle… And, you know, you have turnovers. We have new people… But establish a culture, just like no lotion on CHG…I tell them, you can use the lotion on their feet and, you know, this very dry, little areas …you know, elbows, but don’t slather lotion. Because we have many patients that they want their own lotion. If you put lotion, why this lotion doesn’t smell? You know, so I prefer not to even go there, because I know things going to go awry.	Deviations from CHG treatment steps
**Q6:** Depending on how upset the patient is that you're doing a bath, I mean, it's not going to be perfect, you know, so you might not get every single spot that you would if they weren't moving around like crazy.	Patient factors
**Q7:** … we have a lot of families who like to practice like homeopathic, you know, things and not have, you know, chemicals… so I think it’s challenging.	Patient factors
**Q8:** Where I struggle with CHG treatments is the organizational expectation is that everybody gets a CHG treatment every day. Where I struggle with that is, for the staff to be able to say “every patient needs this every day,” there's, to me there's diff-, there's differences. So like the patient who has a central line, who has a surgical site, they really, really need it. But if it's like everybody needs it, I have a two-week-old who's here with, you know, respiratory infection, who has no lines going into them, nothing, like we're monitoring their breathing.… And if I don't get to this kid, it's okay because he doesn't have anything coming out of him.	Lack of standardized procedures
**Q9:** … change the culture of it because you're, right now it's a little staggered on who is seen as more important, I guess, in that way, but that is something we've discussed recently that we want to make sure it's standardized for all patients …	Lack of standardized procedures
**Q10:** *HCW1*: Because [Nurse Manager] said [CHG] you don't have to rinse off. *HCW2*: Yeah. *HCW3*: But when you put Hibiclens on it… *HCW 1*: You don't have to rinse the Hibiclens off. You're not supposed to. *HCW2*: Hmm. *HCW3*: I thought we, we were supposed to. *HCW4*: No, because you want to leave it on. *HCW2*: Really? *HCW1*: I'm confused now.	Lack of standardized procedures
**Q11:** I go around and check to make sure that they're being used. And I ask the, the techs, actually go around and ask them, are you using the Ziploc bags? Oh, yes.	Monitoring and feedback
**Q12:** Like if it's a baby, if I have to get a weight, I'll do a weight and a bath all at the same time, because they're going to be naked anyway, so it would make sense to do that.	Problem-solving and workarounds
**Q13:** … when I assist the people, they’re, it’s more like I’m giving you the washcloth… So it’s like you’re going to keep scrubbing pretty much until I give you the rinse cloth and say it’s time to rinse… I like while they do their front, I do the back end. So it’s like I know by the time I get done with the back, now it’s going to be time to rinse your front… So I give you the washcloth to rinse your front while I go now back to the back and rinse your back.	Problem-solving and workarounds

### Barriers to compliance

All units experienced some challenges to CHG implementation and ongoing compliance. Barriers spanned all phases of CHG implementation and treatment, and while there was some overlap in the general types of barriers, specific barriers varied across facilities.

### Variations in HCW training

All units described challenges in providing universal, timely and comprehensive training on CHG, leaving gaps in knowledge around how and when to provide CHG treatments. However, the specific ways in which these gaps occurred varied. In Unit A, the IP indicated pre-implementation education did not reach all HCWs and there was reliance on ‘catching up’ people who missed initial sessions through one-on-one instruction. HCWs observed that float staff, occupational therapists, and nursing students, seemed unaware of the CHG policy and procedures.

In Unit B, HCW training was described as a checklist ‘orientation.’ The NM acknowledged HCW treatment competency was not assessed, and after initial rollout, staff were essentially just given a different soap and expected to give the baths properly. The NM also stated training did not cover the benefits, procedures and importance of sufficiently engaging patients/families in CHG treatments.

The NE in Unit C described multiple concurrent projects on the unit (e.g., projects on mobility, immunizations and discharge notes) and the need to balance the educational needs of each project (Q1). This NE acknowledged reviewing project materials quickly in preparation for implementation. Similar factors applied were noted in Unit D, which was in the same institution as Unit C.

### Supply challenges

Challenges due to supplies were common. All units experienced time-consuming spills with the gusseted treatment bags. In addition, some units experienced challenges due to supply shortages, incorrectly-sized supplies and/or supplies stored in multiple locations. Unit A’s stocking schedule was inconsistent with treatment schedules. Linens were stocked after treatments were typically done, and there were occasional linen shortages on weekends. HCWs in Unit A wanted to be good stewards of resources, and the initial use of large bottles of CHG was wasteful, which caused staff discomfort.

In Unit C, CHG-related supplies were stocked in multiple locations on the unit; the CHG pump was occasionally stocked separately from the bottle, and the gusseted bags were stored in the nurse’s station or NM’s office rather than with other supplies. Further, Unit C used indication-based CHG treatments, which resulted in inconsistent need for supplies, and under or overstocking depending on how many patients required CHG treatments in a specific timeframe.

HCWs in Unit D, while seemingly accustomed the CHG treatment practice, described the need to retrieve supplies from multiple locations. They estimated supplies in their own supply room were depleted almost half the time, and they had to go to other supply rooms to get what they needed. Each supply room stocked supplies in different locations, making it difficult for HCWs to quickly find what they needed.

### Inconsistent patient and family education

There was variation reported in how patient/families were educated on CHG before treatments and HCWs, IPs and NMs acknowledged this as a critical step for preventing patient refusals. Some HCWs reported completing patient/family education and others not. In Unit A, written materials were available upon admission for patient/family education. However, HCWs noted that most patients/families did not read the materials, requiring the HCW to spend time explaining the process to them. HCWs stated that they were aware some patients were given CHG baths without receiving any education or providing informed consent. HCWs noted that the unit had been discussing how to improve patient education on CHG.

In Unit B, some HCWs followed procedures and informed and educated patients before giving the CHG treatment. These HCWs felt it was important to prevent waste when bringing supplies to rooms where patients initially refuse a CHG treatment. Other HCWs discussed not providing patient/family education (Q2).

HCWs in Unit C and D made efforts to educate patients/families but were not aware of resources to help. The unit had developed an educational video about CHG treatments that was designed for patients to watch upon admission to the unit. HCWs were not aware the video existed.

### Deviations from CHG treatment steps

HCWs and NMs described both intentional and unintentional deviations from the CHG treatment protocol. These deviations largely occurred due to lack of knowledge or training and a few other unique factors on each unit. HCWs in Unit B intentionally deviated from protocol in their use of new washcloths for each body part or for rinsing because they felt the size of infants and young children made it possible to fold washcloths in quarters and still accomplish the same goal without wasting supplies (Q3). HCWs in Unit B also inconsistently used lotion depending on workload demands or patient behavior. HCWs in Unit B were unaware that CHG should remain on a patient’s skin for at least one minute before rinsing. They stated it would be awkward, difficult and uncomfortable for the patient to have the CHG left on for a minute before rinsing (e.g., due to pediatric patient behaviors or room temperature). The NM also did not know that CHG should be left on the skin for one minute.

HCWs in Unit C described how every worker provided CHG treatments differently (Q4). Some HCWs believed CHG treatments were to be given every 8 hours. Some HCWs did not know lotion should be applied after the treatment and others knew about applying lotion but said long-stay patients complained of dry skin after many days of CHG baths despite use of lotion.

The NM in Unit D asked HCWs to deviate from protocol and not use lotion because they did not have any CHG compatible lotion on their unit. For this reason, she also tried to establish a culture that no lotion is used after CHG treaments (Q5).

### Patient factors

Most units experienced challenges with patient factors that occasionally prevented fully compliant treatments. In Unit A, HCWs described patient factors such as independence, large size, poor skin integrity or preferences affected CHG use. Independent patients were encouraged to use CHG, but some chose not to or to not bathe entirely, and some took independent showers where they may or may not use CHG or use it improperly.

HCWs in Unit B frequently mentioned patient characteristics as a barrier. Patient age, developmental stage and behaviors, parent involvement and preferences sometimes influenced whether bathing occurred (Q6). Toddler behaviors made bathing challenging. Pre-teens were generally less interested in bathing but old enough to voice and have their preference respected; teenagers showered independently where HCWs could not ensure compliance with the protocol. CHG soap also did not seem to reduce body odor experienced by teenagers. Bathing was reported to be a low priority to parents and children in this setting, and children who were not feeling well or had a rough night may not want to bathe. Further, some parents refused baths, gave baths, or requested/used other soap (Q7).

HCWs in Units C and D also reported issues with patient refusals when not feeling well, having pain or when daily bathing did not align with their usual bathing practices (e.g., weekly bathing at home). The HCWs did feel if they provided rationale for the use of CHG, it could increase compliance with the protocol.

### Documentation

Documentation was a challenge in some units. In Unit B, the electronic health record (EHR) was slow to load. The EHR also produced a worklist for daily tasks with red checks that turned green when complete. HCWs would check the CHG treatment off the list even if it had not been done in an effort to remove all tasks before the end of their shift. This prevented communication to the next shift that the CHG treatment still needed to be done. HCWs in Unit D reported documenting in the EHR was challenging and that they had to document in multiple locations to avoid repercussions of inaccurate or incomplete documentation.

### Lack of standardized procedures

There were multiple ways in which procedures were not standardized across units, creating gaps and potentially influencing compliance. Some units did not provide CHG treatments to all patients, some units did not standardize soaps leaving HCWs to make choices about which to use, and others did not standardize training to ensure HCWs got the same message about how to give a CHG treatment.

In Unit A, non-CHG soaps were available during implementation, and some staff preferred and used non-CHG (e.g., because of perception of CHG as sticky).

The IPs and NM recognized that on Unit B that CHG treatments were not a priority for all patients and that standardization of the CHG bathing process across patients was needed. The NM struggled with telling staff that every patient should have a CHG treatment, particularly if they did not have lines or surgical sites and work demands were high (Q8). Also, the NM felt that a challenge with this units was that they ‘take everybody who doesn’t fit anywhere else,’ which meant they had many different types of patients and standard of care was different for each type.

The IP leader of Units C and D recognized the challenge of following a different process for CHG treatments organization-wide, especially for float staff. The nursing director also recognized the wide variation in how CHG treatments were given and was committed to standardizing the process (Q9), EHR documentation and HCW training and education.

In Unit D, where indication-based treatments were used, there was considerable variation in the ways HCWs described giving treatments and confusion over some of the parameters. Some staff would use non-CHG impregnated disposable washcloths (Coloplast^®^) together with the CHG because they were recommended for use by wound care, others would use the recommended gusseted plastic bags, and others would use basins. The NM indicated there was not a clear standard of practice with basins; some staff used them but cleaned them with antiseptic wipes first, others used them but did not dry them, and others threw them away. Some HCWs were confident that the CHG should be rinsed off, and others were confident it should remain on the skin (Q10). Throughout the discussion of giving treatments, HCWs on Unit D were actively clarifying procedures and giving tips to one another on how to best give the treatment during the focus group. They further mentioned that each individual had their own unique way of giving a treatment, and they often had to clear or confirm their practices with the NM.

### Facilitators of compliance

Each unit had some examples of practices that may have influenced the success of CHG implementation or use. Participants did not necessarily identify these practices as facilitators; they discussed these practices in the context of initial or ongoing implementation to support the use of CHG. These work system facilitators were put in place by NMs. Individual HCWs devised limited strategies to improve CHG compliance as well.

### Communication

Most unit NMs described using multimodal communication strategies to train staff and facilitate initial and ongoing CHG implementation. The NM in Unit A cascaded initial communication about CHG treatment implementation down from unit councils to HCWs. The NM spent extensive time and repeated efforts to communicate about the initiative by scheduling multiple long meetings, followed by weekly check-ins and concluding with information sharing as needed in daily huddles. Initial education was also well organized and systematic, allowing time for staff discussion and comment on training needs and materials. Unit A also had front-line HCW ‘champion,’ which the NM believed was largely responsible for unit success.

In Unit B, the NM used weekly newsletters and posted signs to get messages to staff about CHG treatments that included rationale for using CHG, making it clear nurses are accountable when baths are delegated and sharing compliance data (albeit delayed).

The NEs in Units C and D held monthly meetings, shared information during daily huddles, used email, posted signs, and attempted to touch base with everyone. There was also a suggestion box that could be used for anything staff wanted to communicate on the unit.

### Monitoring and feedback

Each unit engaged in monitoring and feedback to share the current state and assess gaps in performance and/or need for additional training. Unit B used Kamishibai cards (K-cards) [30], as a means of providing “instant feedback” through a unit-based, patient-focused case presentation process.

The NE on Unit C did frequent observation of staff work when in patient rooms or on the unit, communicating any deviations from recommended practices in the moment. If she felt staff did not understand or would continue to work around the suggestion, she might continue monitoring them and report the behaviors to the next level if not corrected. She also monitored plastic bag stock levels to judge CHG treatment compliance (Q11); if a patient developed an infection, she would audit the chart and look to see, among other things, that the patient received their CHG treatment.

The NE in Unit D used casual observations to monitor staff practices. For example, she occasionally helped HCWs give baths to do direct observation and provide real-time feedback and one-on-one teaching. The NE indicated the EHR was not the best method for monitoring treatments because it requires an order to be placed and for the nurse to ‘go out of their way’ to put it in. She felt then that there were more CHG treatments given than actually documented.

### Problem-solving and workarounds

Staff across all units developed specific strategies to work around barriers and improve compliance. To overcome supply challenges, Unit B made bath bags, which contained everything needed to give a CHG treatment. To prevent spills, HCWs in Unit A placed the gusseted bag inside a basin, using only one washcloth in the bag at a time or propping the bag between objects like soap bottles to keep it from falling over. To address wrong-sized supplies, the IP on Unit A ordered smaller bottles of CHG to prevent waste.

To improve patient acceptance of CHG treatment without creating additional work, HCWs in Unit B bundled bathing tasks with others, such as wound care or getting weights (Q12). HCWs also prioritized baths for patients with lines or upcoming procedures and coordinated their treatments with the patient’s schedule and making repeated requests to the patient about bathing. HCWs in Units A and D explained the purpose of CHG to patients in non-medical language to prevent refusals and HCWs in Unit D encouraged the use of CHG despite preferences for the patient’s own soap by providing rationale for using CHG. HCWs in both Units C and D ensured independent patients received education about CHG. Some HCWs in Unit A used an aided-independence strategy to prevent inappropriate patient use of CHG by pacing and controlling how it is applied and rinsed. (Q13).

To improve training gaps for float staff, one HCW in Unit A provided on-the-spot training by locating and sharing a CHG training video on you-tube.

## Discussion

Using a multiple case design and multiple data collection methods, we explored factors associated with implementing daily CHG treatment intervention in non-ICU settings. We found variation across cases in CHG treatment practices, compliance and skin CHG, highlighting important considerations for implementation of this treatment non-ICU settings. Treatments lasted the longest on Unit D (neuroscience unit); this type of patient may pose a number of challenges related to the amount of time necessary to provide personal care. Units C and D had the lowest proportion of CHG-compatible lotion use. HCWs in these units reported an unawareness of CHG compatible lotions and were actually encouraged not to use lotion on Unit D as it was not stocked.

Data on CHG concentration on the skin showed that Unit B had the lowest proportion of patients with detectable CHG concentration for the two swab collection times for all anatomical sites, while Units C and D (combined) had the highest proportion. The low concentration for Unit B could be partly explained by the HCW’s lack of awareness to leave CHG on a patient’s skin for one minute to ensure effectiveness. In addition, using a single washcloth on the patient’s entire body might also explain low skin CHG concentrations.

The focus group and interview data show variation in initial implementation practices, as well as how CHG treatment practices are carried out by HCWs. These variations are related to differences in organizational education and training practices, feedback and monitoring practices, patient education or information about CHG treatments and patient preferences and general unit patient population differences. All four cases reported some difficulty with obtaining CHG supplies and using the gusseted bag. However, Unit B was better able to overcome these issues by developing workaround strategies, such as propping up gusseted bags in basins or developing a supply kit for bathing. Further, all four units described challenges with unique patient needs and preferences, which prevented following recommended practices. Nevertheless, the specific challenges were different across units depending on their unique patient population (e.g., using a single washcloth by staff at Unit B, pediatric hospital).

Integrating all sources of data, Case A may demonstrate relatively maximum compliance among the four cases. Case A had the highest proportion of observations in compliance with recommended practices, and evidence of CHG on the skin was higher than case B, although somewhat lower than Cases C and D. HCWs from Case A also reported fewer barriers to performing CHG treatments. Case B also had relatively high compliance, but focus groups revealed multiple barriers, most related to their unique pediatric patient. Case B had the lowest levels of CHG detected on the skin; whether this is linked to the practice of immediate rinsing of CHG is an important research question to explore. Cases C and D had lower levels of compliance with multiple aspects of the CHG treatment, yet high levels of CHG detected on the skin. Participants in Cases C and D also reported more confusion about the procedure and therefore there was overall variation from person-to-person in treatment practices. Using indication-based decisions about who should get a CHG treatment and the use of different forms of soap for different types of patients were also sources of confusion.

Case A had a systematic implementation process and the most recent implementation. This could explain the high levels of compliance with the CHG treatment process and the observation that, overall, Case A HCWs seemed knowledgeable about how to complete each step of the procedure. For Case A, pre-implementation education was well-organized, with formal presentation of the CHG treatment procedures at multiple staff levels and to multiple stakeholders with opportunities to ask questions. Unlike other cases, uniform training and the fact that this was the only unit using CHG in the hospital likely ensured less confusion in completing some steps of the CHG treatment. Indeed, conducting initial and ongoing training is an essential implementation strategy [31, 32]. Hence, for successful implementation, facilities need to devote enough time and resources to develop educational materials and provide education or training to staff and other stakeholders about new procedures [31, 32]. Less upfront time spent educating staff or getting buy-in and varied training and staff unawareness about training materials could explain lower compliance in Cases C and D. In addition, patients at Cases C and D had to have a certain indication (e.g., an intravenous line) to receive CHG treatment, which may have created confusion for HCWs.

Although education is important, implementation of complex interventions requires more extensive changes than education or training alone [33]. Other implementation strategies used in Case A, including enhancing motivation (identification and use of champions), adequate resources, continuous improvements and increasing skill development (coaching by researchers) and removing environmental constraints (seeking and obtaining buy in of frontline staff regarding CHG use) [33] have been documented in the literature as important determinants of successful implementation [34].

A major strength of our study is the use of multiple complementary methods in cross-case analysis design. This enabled us to describe factors identified by multiple means of data collection that influence the implementation of CHG treatment [35]. With this approach, we were able to systematically relate qualitative data with quantitative measures of compliance. Moreover, exploring factors associated with implementing the CHG treatment intervention in non-ICU settings is a major contribution, as recent literature reports that CHG treatments are primarily performed in the ICU [36, 37]. Since the inception of this project, a randomized clinical trial (RCT) has shown that among non-ICU patients, daily CHG bathing plus nasal decolonization for MRSA carriers reduced MRSA or VRE clinical cultures and all-cause bloodstream infections only in patients that had medical devices [8]; a recent review of this trial has addressed questions, such as who, what, where, when, and why, regarding CHG-based decolonization [38]. Some facilities might consider implementing CHG treatment hospital-wide, and others might focus only on patients with medical devices.

This study has some limitations. First, there is a possibility of bias introduced due to convenience sampling for focus groups and interviews. We attempted to address this by involving HCWs from both morning and evening shifts in focus groups. However, we acknowledge that our sample of 4–6 HCWs per unit may not be representative of all the HCWs on a given unit. We complemented our qualitative data collection by interviewing individuals with leadership roles directly involved in implementing CHG treatment (e.g., nurse managers, nurse educators, infection preventionists). Another limitation is the relatively small sample size of swab data from Cases C and D; however, the data support the exploratory nature of the project. Finally, the various data sources were collected concurrently, limiting the opportunity to use observation and swab data to guide questions in focus groups or interviews. Therefore, while the qualitative data provided insights into potential variation in compliance, the concurrent design does not allow for direct triangulation between the data sources.

## Conclusion

This project highlighted variation in CHG implementation across four different non-ICU settings and provided insights into possible explanations for this variation and its effects on compliance. Organizations planning to implement CHG treatments in non-ICU settings need to ensure organizational readiness and buy-in and need to consider delivering systematic training or education across all levels of staff and various stakeholders. The presence of unit champions may strengthen unit-wide buy-in and support among HCWs conducting CHG treatments. Further, considering clear and systematic implementation policies across patients and units may help reduce potential confusion about treatment practices and variation across HCWs. Building structures for feedback and ongoing monitoring of treatment compliance is important. Communication between units conducting CHG implementation is essential because this allows units to share their experience and learn from each other. Finally, factors specific to particular settings (e.g., patient type) need to be carefully considered and procedures developed to ensure compliance given unique patient challenges.

## Supporting information

S1 File Interview guide for infection preventionist. (PDF)

S2 File Interview guide for nurse manager or assistant. (PDF)

S1 Data (XLSX)
