# Supplementary material for: Implementing daily chlorhexidine gluconate treatment for the prevention of healthcare-associated infections in non-intensive care settings: A multiple case analysis
Source: PLoS One. 2020 Apr 24;15(4):e0232062. doi: 10.1371/journal.pone.0232062 (PMC7182260; doi:10.1371/journal.pone.0232062)
Supplement: S1 File — (PDF) [file pone.0232062.s001.pdf]

INTERVIEW GUIDE FOR INFECTION PREVENTIONIST
I-CARE PROJECT

Interviewee signature/initials:______________________ Date: __________________

Organization: ______________________________ Begin time: _____________

Introduction:

Thank you for taking the time to meet with me today. Throughout this interview, I will refer to CHG treatments rather than baths. I would like to talk with you about your experiences with the implementation of daily CHG treatments in your facility. As a quality improvement project, the information obtained will help us capture lessons that can be used in future interventions.

The interview will take about 30-45 minutes. To ensure I don't miss any of your comments, may I audio record our conversation? (If yes, turn on two recorders.) If you inadvertently share any confidential information about yourself or patients, that information will be deleted from the recording and any documents created.

Your responses to all questions in this interview will be kept anonymous and confidential. Any information we include in write ups or publications will not identify you as the respondent. You don't have to talk about anything you don't want to and you may end the interview at any time.

Do you have any questions about what I have just explained?

Are you still willing to participate in this interview?

Questions:

1. To better understand all you have to do, could you please briefly describe your job duties and responsibilities? (brief description of your job)
2. How much of a priority is the CHG treatment intervention compared to all your duties? Please explain.
3. Generally speaking, how do you communicate evidence-based initiatives to your staff?
4. Specifically, how was the CHG intervention communicated to your staff? What was unique about communicating the CHG treatment intervention?
5. How do you communicate the CHG treatment policies and procedures to new staff?

6. How did you plan for the CHG intervention? What activities occurred? Who was involved?
7. Please describe the training you received about CHG treatment prior to implementation. Who led it? What did it entail?

8. How do you monitor that CHG treatment is done properly or that staff comply with the treatment protocol?
   a. Do you monitor CHG treatment supplies? How do you do that?
   b. Do you monitor infection rates? How?
   c. Do you conduct or oversee some form of documentation auditing or chart review?
   d. Do you do direct observation of CHG treatments?
   e. Do you share results with staff? (If so) How (in what format or mechanism) and when do you share it?
9. Do you monitor or get feedback on whether staff have all the supplies/equipment to successfully carry out the CHG treatment? (If so) How?

10. What are factors that make it easy for staff to perform CHG treatments?
11. What factors interfere with staff's abilities to appropriately and efficiently perform CHG treatments?
12. What do you do when you observe staff giving a CHG treatment who need assistance?

13. What challenges did you face in implementing CHG treatment?
14. What are some of your success stories since implementing CHG treatment?
15. Thinking about the CHG treatment implementation, is there anything you would do differently if you were to implement it elsewhere [in the hospital/in another facility]? What would you replicate?

16. [ASK this question only if implementation was not hospital-wide] What do you think about implementing CHG treatment hospital-wide?
17. Do you have any questions for me? Is there anything more you would like to add or tell me about?
18. How do you feel about adding CHG treatment to the order set?

End time: _____________

Is it ok, to contact you again if I have extra questions or seeking clarification?

THANK YOU FOR YOUR TIME.

1. Items that came up in this interview that are worth following up in the next interview
   - **a.** ________________
   - **b.** ________________
   - **c.** ________________
   - **d.** ________________
   - **e.** ________________
   - **f.** ________________
